# A body shape index combined with the triglyceride-glucose index for cardiovascular risk prediction in overweight and obese Chinese adults

**DOI:** 10.3389/fcvm.2026.1712006

**Published:** 2026-04-29

**Authors:** Xing Li, Xiangmei Li, Fen Liu, Long Yang, Yining Yang, Yuling Wang

**Affiliations:** 1 Health Center, First Affiliated Hospital of Xinjiang Medical University, Urumqi, China; 2 State Key Laboratory of Pathogenesis, Prevention and Treatment of High Incidence Diseases in Central Asia, Department of Cardiology, First Affiliated Hospital of Xinjiang Medical University, Urumqi, China; 3 Thyroid and Breast Surgery, General Hospital of Xinjiang Military Region, Urumqi, China; 4 Department of Cardiology, People’s Hospital of Xinjiang Uyghur Autonomous Region, Xinjiang, China; 5 Xinjiang Key Laboratory of Cardiovascular Homeostasis and Regeneration Research, Urumqi, Xinjiang, China

**Keywords:** a body shape index, cardiovascular disease, insulin resistance, obesity, overweight, triglyceride-glucose index

## Abstract

**Background:**

Overweight and obesity are major modifiable risk factors for cardiovascular disease (CVD). A body shape index (ABSI) reflects abdominal adiposity independently of body mass index (BMI), whereas the triglyceride–glucose (TyG) index is a validated surrogate marker of insulin resistance (IR). Therefore, the present study aimed to evaluate the independent and combined predictive value of ABSI and the TyG index for CVD risk among overweight and obese Chinese adults.

**Methods:**

This retrospective cohort study included 920 overweight or obese adults aged 40–80 years who were free of CVD at baseline and underwent routine health examinations at the First Affiliated Hospital of Xinjiang Medical University from January 2019 to October 2019. Baseline anthropometric indices, including BMI, waist circumference (WC), waist-to-height ratio (WHtR), body roundness index (BRI), and ABSI, as well as laboratory parameters, were assessed at enrollment. Participants were followed through October 2024. Logistic regression was used to examine associations between indices and incident CVD. Restricted cubic spline (RCS) analysis assessed nonlinear relationships. Predictive performance was evaluated using receiver operating characteristic (ROC) curves and the area under the curve (AUC).

**Results:**

Among 920 participants, 310 (33.7%) developed CVD during follow-up. Logistic regression showed that both the TyG index (OR: 2.093; 95% CI: 1.532–2.889) and ABSI (OR: 1.791; 95% CI: 1.432–2.256) were independently associated with incident CVD after multivariable adjustment. RCS analysis demonstrated a nonlinear S-shaped association between the TyG index and CVD (*P* for nonlinearity = 0.006), while ABSI exhibited a linear relationship (*P* for nonlinearity = 0.390). ROC analysis indicated that the TyG index (AUC = 0.636), ABSI (AUC = 0.634), WC (AUC = 0.611), triglycerides (TG; AUC = 0.616), and BRI had moderate predictive ability. The combined model of TyG + ABSI achieved the highest predictive accuracy (AUC = 0.679, 95% CI: 0.644–0.715).

**Conclusion:**

Both ABSI and the TyG index were independently associated with incident CVD in overweight and obese Chinese adults. Their combined use demonstrated a modest improvement in discriminative performance compared with either index alone, suggesting potential complementary value for cardiovascular risk assessment.

## 1 Background

Cardiovascular disease (CVD) is a significant global public health issue, causing substantial mortality and disability annually (1). In China, the burden of CVD has increased steadily in recent decades, largely attributable to population aging and lifestyle transitions characterized by reduced physical activity, increased sedentary behavior, and dietary shifts toward high-calorie, high-fat, and high-sodium foods. Statistics show that deaths from CVD, including coronary heart disease and stroke, account for over 40% of all disease-related deaths among Chinese residents (2). Hypertension, dyslipidemia, and abnormal blood glucose play critical roles in CVD development. Among the many risk factors for CVD, overweight and obesity are modifiable risk factors. In recent years, the prevalence of overweight and obesity among adults in China has risen significantly, with nearly half of middle-aged and elderly people estimated to be classified as overweight or obese (3, 4).

Traditionally, body mass index (BMI) has been the most commonly used anthropometric indicator for defining overweight and obesity, with the advantages of simplicity and universal applicability. However, BMI cannot distinguish between fat mass and lean mass, nor can it account for fat distribution (5). Studies have shown that individuals with similar BMI values may exhibit markedly different CVD risks depending on the extent of visceral fat accumulation (6). To overcome these limitations, alternative anthropometric indices have been proposed. Waist circumference (WC) is frequently used as a marker of central obesity, whereas waist-to-height ratio (WtHR) has been suggested as a simple yet powerful predictor of cardiometabolic risk. WtHR is based on the principle that WC should be less than half of an individual's height to minimize health risks (7, 8). The body roundness index (BRI), derived from a geometric model incorporating WC and height, aims to estimate body fat percentage and visceral adiposity more accurately than BMI (9). Although these indices improve the assessment of fat distribution, their predictive ability for cardiovascular outcomes has been inconsistent across different studies and populations. In recent years, a novel anthropometric measure, a body shape index (ABSI), has been introduced as an index specifically designed to assess abdominal obesity independently of BMI. ABSI is calculated using WC adjusted for height and weight, thereby minimizing collinearity with BMI (10). Studies have demonstrated that ABSI is significantly associated with all-cause mortality, cardiovascular events, and metabolic diseases (11, 12). ABSI provides unique information on body shape and fat distribution that cannot be captured by BMI or WC alone (13).

The triglyceride-glucose (TyG) index, derived from fasting triglycerides (TG) and fasting plasma glucose (FPG), has been validated as a simple and reliable surrogate marker of insulin resistance (IR) (14). Studies have demonstrated that elevated TyG index values are associated with both incident CVD and adverse prognosis (15). Given that ABSI and the TyG index capture different but complementary aspects of cardiovascular risk—namely, body shape and fat distribution vs. metabolic status and IR—their combined application may enhance risk prediction more effectively than either measure alone, particularly in overweight and obese populations where excess adiposity and metabolic abnormalities frequently coexist (16). A study in the United States reported that the combined use of ABSI and the TyG index provided better predictive ability for cardiovascular mortality compared with TyG alone or other anthropometric indices (12). In addition, a study in a Chinese population demonstrated that the combination of ABSI and the TyG index was associated with an increased risk of stroke in the general population (17). However, several important knowledge gaps remain. First, existing studies in Chinese populations have not systematically compared the predictive performance of the ABSI–TyG combination with that of the TyG index combined with other commonly used anthropometric indicators, such as BMI, BRI or WtHR, and the incremental predictive value of this combined index has not been quantitatively evaluated. Second, prior studies have primarily focused on general populations or mortality outcomes, with limited evidence regarding incident CVD events among individuals at elevated cardiovascular risk. Consequently, whether integrating anthropometric and metabolic indices can meaningfully improve the prediction of incident cardiovascular disease in high-risk Chinese populations remains insufficiently explored.

Therefore, the present study aimed to evaluate whether the combined use of ABSI and the TyG index improves the prediction of incident cardiovascular disease beyond either index alone among overweight and obese Chinese adults. This research is expected to provide new insights for risk stratification strategies, support the development of simple yet effective tools in clinical practice, and contribute to the prevention and early detection of CVD in high-risk populations.

## 2 Methods

### 2.1 Study design and population

This study employed a retrospective cohort design and adhered to the Strengthening the Reporting of Observational Studies in Epidemiology (STROBE) guidelines. The participants were overweight or obese patients who underwent routine health examinations at the Health Care Center of the First Affiliated Hospital of Xinjiang Medical University between January 2019 and October 2019. The study protocol was approved by the Ethics Committee of the First Affiliated Hospital of Xinjiang Medical University (Approval No. K202403-59). Owing to the retrospective nature of the study, informed consent was waived. Prior to analysis, all personal identifiers were removed, and the dataset was anonymized to ensure participant confidentiality. Data were accessed and analyzed in a de-identified form in accordance with institutional ethical guidelines.

The inclusion criteria were as follows: (1) age between 40 and 80 years; (2) no prior history of CVD; and (3) overweight (BMI ≥ 25), obesity (BMI ≥ 30), or abdominal obesity (WC ≥ 85 cm for women and WC ≥ 90 cm for men). (3) complete ABSI and TyG metrics. The exclusion criteria were: (1) presence of established or newly diagnosed CVD during the examination; (2) history of other structural heart diseases, including congenital heart disease, hypertrophic cardiomyopathy, valvular heart disease, and pericardial disease; (3) diagnosis of malignancy (including but not limited to lung, gastric, hepatic, colorectal, or breast cancer) or undergoing chemotherapy or radiotherapy; (4) thyroid diseases, including hyperthyroidism, hypothyroidism, Hashimoto's thyroiditis, or thyroid cancer; (5) abnormal liver function or renal impairment, included alanine aminotransferase (ALT) or aspartate aminotransferase (AST) > 3 times the upper limit of normal, and estimated glomerular filtration rate (eGFR) < 60 mL/min/1.73m^2^; (6) history of major surgery within the past year.

### 2.2 Clinical data collection

All clinical, biochemical, and anthropometric data were collected at baseline, at the time of enrollment. Trained physicians collected baseline medical histories using standardized electronic questionnaires. The collected information included general participant characteristics (gender, age), risk factors (smoking status, family history of cardiovascular or cerebrovascular disease, history of diabetes, and history of hypertension). Physical examinations were conducted following standardized procedures and included measurements of height, weight, waist circumference, and blood pressure. Fatty liver status was also assessed at baseline by abdominal ultrasonography. According to the ultrasound findings, fatty liver was classified as mild, moderate, or severe. Because only three participants were classified as having severe fatty liver in this study, the moderate and severe groups were combined for subsequent analyses. For laboratory tests, professional nurses collected fasting venous blood samples (at least 8 h of fasting) and immediately transported them to the laboratory. Complete blood counts were measured using an XN-1000 automated hematology analyzer (Sysmex Corporation, Japan). Fasting blood glucose (Glu), triglycerides (TG), total cholesterol (TC), directly measured low-density lipoprotein cholesterol (LDL-C), high-density lipoprotein cholesterol (HDL-C), creatinine, aspartate aminotransferase (AST), and alanine aminotransferase (ALT) levels were analyzed using a DXC800 automated biochemical analyzer (Beckman Coulter, USA). The estimated glomerular filtration rate (eGFR) was calculated using the Modification of Diet in Renal Disease (MDRD) formula (18). The TyG index is calculated using the following formula: TyG index = ln (TG(mg/dL) × FPG(mg/dL)/2) (15).

### 2.3 Anthropometric measurements

In this study, several anthropometric indices were calculated based on height (m), weight (kg), and WC (m), including ABSI, BMI, WtHR, and BRI. The specific formulas were as follows: (1) BMI = weight/height^2^; (2) WtHR = WC/height; (3) BRI = 364.2 − 365.5 × sqrt[1 − (WC/2π)^2^/(height/2)^2^](9); (4) ABSI = WC/[BMI^(2/3)^ × height^(1/2)^] (10). According to BMI, patients were classified as overweight (BMI ≥ 25) or obese (BMI ≥ 30). Abdominal obesity was defined according to the International Diabetes Federation (IDF) criteria as WC ≥ 85 cm for women or WC ≥ 90 cm for men (19). Importantly, individuals who met the criteria for abdominal obesity but had a BMI < 25 kg/m^2^ were classified as abdominal obesity group. Thus, BMI-defined overweight/obesity and WC-defined abdominal obesity were treated as partially overlapping but non-mutually exclusive categories in descriptive analyses and regression models.

### 2.4 Follow-up

Patients at the Health Care Center of the First Affiliated Hospital of Xinjiang Medical University underwent routine annual health examinations. This study recorded physical examination results from each visit during the follow-up period from January 2020 to October 2024, spanning 1–5 years after the initial examination. New-onset CVD was identified based on electronic medical records (visits to our hospital after the initial physical examination) and clinical interviews. Diagnostic criteria for new-onset CVD included coronary heart disease (CHD), myocardial infarction (MI), stroke, heart failure (HF), and other major cardiovascular events (e.g., hospitalization for any cardiovascular event or cardiovascular death). Participants were defined as having new-onset CVD if they experienced any of these outcomes during follow-up.

### 2.5 Statistical analysis

Statistical analyses were performed using R software (version 4.4.1). Missing covariates were handled using multiple imputation. Covariates with missing proportions exceeding 20% were excluded from the imputation process. Multiple imputation was conducted using the “mice” package, applying the predictive mean matching (PMM) method under the assumption that data were missing at random (Supplementary Table 1). Continuous variables were assessed for normality using the Shapiro–Wilk test. Continuous variables were expressed as mean ± standard deviation (*x* ± *s*), and comparisons between two groups were performed using Student's *t*-test. Categorical variables were presented as frequencies (percentages), and comparisons between groups were performed using the chi-squared (*χ*^2^) test. Logistic regression analysis was used to assess the relationship between the TyG index and physical measurement indicators and the incidence of CVD. Variables that were clinically important or showed an association with CVD in univariable analyses (*P* < 0.05) were retained in multivariable logistic models to minimize residual confounding. To explore potential nonlinear associations, restricted cubic spline (RCS) regression was applied within the logistic regression framework using the “rms” package. The TyG index was modeled using four knots, placed at the 5th, 35th, 65th, and 95th percentiles of its distribution. The median value of the TyG index was used as the reference point. Receiver operating characteristic (ROC) curves were plotted, and the predictive efficacy of each index for CVD was evaluated using the area under the curve (AUC), sensitivity, specificity, positive predictive value (PPV), negative predictive value (NPV), and Youden's index. To assess the incremental predictive value of composite indices, logistic regression models were constructed by combining the TyG index with ABSI, WtHR, BRI, and BMI. ROC curves and AUC values were generated based on the predicted probabilities from these models.

## 3 Results

### 3.1 Baseline characteristics of participants

**Figure 1:**
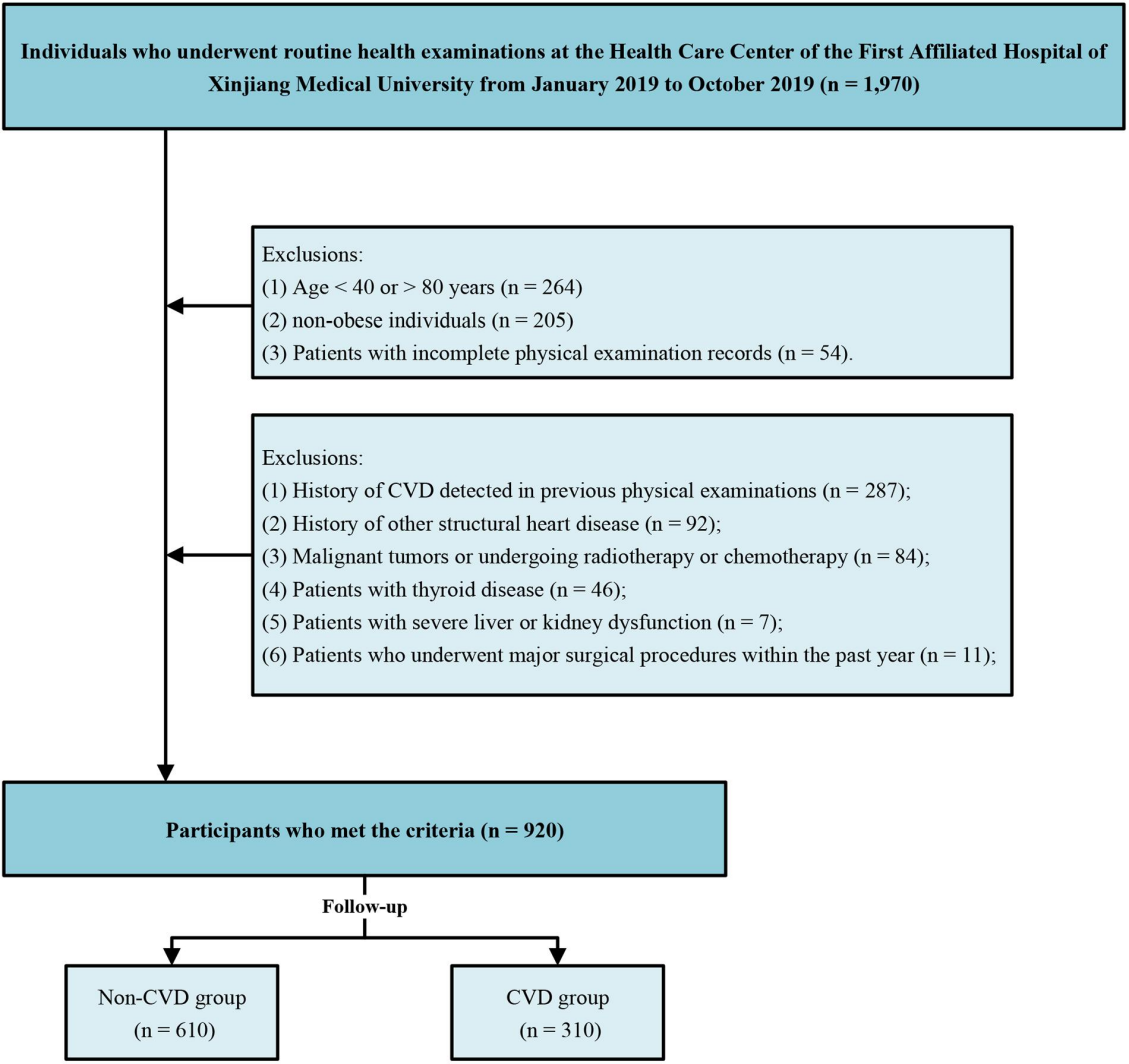
Flow chart of study inclusion-exclusion criteria.

In the overall dataset of this study, 54 participants (2.7%) were lost to follow-up. After applying inclusion and exclusion criteria, a total of 920 overweight or obese patients were enrolled in this study (Figure 1). Participants were categorized into CVD group (*n* = 310, 33.7%) and non-CVD group (*n* = 610, 66.3%) based on the incidence of cardiovascular disease (CVD) during follow-up. No cardiovascular deaths occurred during the follow-up period.

The mean age of participants was 61.3 ± 8.4 years, including 787 men (85.5%) and 133 women (14.5%). Significant differences were observed between the two groups in terms of age, hypertension, diabetes, family history of cardiovascular or cerebrovascular disease, systolic blood pressure, and diastolic blood pressure (*P* < 0.05). The proportion of abdominal obesity was higher in the CVD group, whereas the proportions of overweight/obesity were lower compared with the non-CVD group (*P* < 0.05).

Regarding anthropometric parameters, there were significant differences between the two groups in height, weight, ABSI, WtHR, BRI, and WC (*P* < 0.05). For laboratory measurements, significant differences were found in the TyG index, Glu, HDL-C, TG, eGFR, white blood cell count (WBC), and neutrophils (*P* < 0.05). No significant differences were observed between the two groups in smoking, drinking, hepatic steatosis, BMI, TC, LDL-C, albumin, lymphocytes, ALT, and AST (*P* > 0.05) (Table 1).

**Table 1:** Baseline characteristics of participants grouped according to incidence of CVD.

Characteristics	Total	non-CVD group	CVD group	Statistic	*P*-value
	(*n* = 920)	(*n* = 610)	(*n* = 310)		
Basic clinical information					
Age, years	61.3 ± 8.4	59.4 ± 7.8	65.0 ± 8.2	−9.904	<0.001
Sex				3.414	0.065
Male	787 (85.5)	512 (83.9)	275 (88.7)		
Female	133 (14.5)	98 (16.1)	35 (11.3)		
Hypertension	591 (64.2)	353 (57.9)	238 (76.8)	31.161	<0.001
Diabetes	251 (27.3)	135 (22.1)	116 (37.4)	23.451	<0.001
Smoking	340 (37.0)	238 (39.0)	102 (32.9)	3.04	0.081
Drinking	358 (38.9)	251 (41.1)	107 (34.5)	3.529	0.060
Family CVD history	265 (28.8)	135 (22.1)	130 (41.9)	38.351	<0.001
Hepatic steatosis				1.933	0.380
No	330 (35.9)	213 (34.9)	117 (37.7)		
Mild	520 (56.5)	354 (58.0)	166 (53.5)		
Moderate or severe	70 (7.6)	43 (7.0)	27 (8.7)		
Abdominal obesity	670 (72.8)	412 (67.5)	258 (83.2)	24.766	<0.001
Overweight or obesity	747 (81.2)	515 (84.4)	232 (74.8)	11.754	<0.001
SBP, mmHg	131.4 ± 17.6	129.8 ± 16.5	134.5 ± 19.2	−3.679	<0.001
DBP, mmHg	80.6 ± 10.8	81.3 ± 10.9	79.4 ± 10.4	2.554	0.011
Physical measurement indicators					
Height, cm	171.1 ± 6.8	170.6 ± 7.4	172.0 ± 5.5	−3.238	<0.001
Weight	77.7 ± 9.1	77.1 ± 8.4	78.9 ± 10.3	−2.642	<0.001
ABSI	0.080 ± 0.007	0.079 ± 0.007	0.081 ± 0.007	−5.586	<0.001
WtHR	0.54 ± 0.05	0.53 ± 0.05	0.54 ± 0.04	−2.4	0.017
BRI	4.19 ± 0.93	4.13 ± 0.96	4.32 ± 0.87	−2.920	<0.001
BMI, kg/m^2^	26.5 ± 2.3	26.4 ± 1.6	26.7 ± 3.3	−1.179	0.239
Waist, cm	92.4 ± 7.1	91.6 ± 7.3	94.0 ± 6.5	−5.143	<0.001
Laboratory test indicators					
TyG index	8.93 ± 0.57	8.85 ± 0.56	9.09 ± 0.57	−6.154	<0.001
Glu, mmol/L	5.63 ± 1.75	5.45 ± 1.58	5.99 ± 1.99	−4.158	<0.001
TC, mmol/L	4.93 ± 1.12	4.98 ± 1.06	4.83 ± 1.23	1.783	0.075
HDL-C, mmol/L	1.18 ± 0.25	1.19 ± 0.25	1.15 ± 0.26	2.328	0.020
LDL-C, mmol/L	3.30 ± 0.87	3.33 ± 0.80	3.24 ± 1.00	1.371	0.171
TG, mmol/L	1.99 ± 1.29	1.89 ± 1.21	2.18 ± 1.42	−3.111	<0.001
Albumin, g/L	44.2 ± 2.4	44.2 ± 2.4	44.1 ± 2.4	0.722	0.471
eGFR, mL/min/1.73m^2^	79.1 ± 23.0	81.5 ± 24.1	74.6 ± 19.8	4.651	<0.001
WBC, 10^9/L	6.46 ± 1.54	6.37 ± 1.51	6.63 ± 1.59	−2.401	0.017
Neutrophil, 10^9^/L	3.53 ± 1.06	3.48 ± 1.04	3.63 ± 1.10	−2.018	0.044
Lymphocyte, 10^9^/L	2.18 ± 0.67	2.17 ± 0.66	2.21 ± 0.67	−0.771	0.441
ALT, U/L	26.1 ± 14.4	26.1 ± 14.2	26.3 ± 14.9	−0.264	0.792
AST, U/L	24.9 ± 9.2	24.5 ± 8.8	25.8 ± 9.8	−1.893	0.059

### 3.2 Association analysis between the TyG index and physical measurement indicators to incident CVD

Logistic regression analysis showed that the TyG index (OR: 2.113; 95% CI: 1.653–2.720, *P* < 0.001), BRI (OR: 1.007; 95% CI: 1.001–1.012, *P* = 0.030), and ABSI (OR: 1.818; 95% CI: 1.473–2.256, *P* < 0.001) were significantly associated with the presence of CVD in obese patients. After adjustment for sex, age, hypertension, diabetes, family history of cardiovascular or cerebrovascular disease, systolic blood pressure (SBP), diastolic blood pressure (DBP), WBC, eGFR, neutrophils, and HDL-C, both the TyG index (OR: 2.093; 95% CI: 1.532–2.889, *P* < 0.001) and ABSI (OR: 1.791; 95% CI: 1.432–2.256, *P* < 0.001) remained independently associated with incident CVD. No significant associations were observed for WtHR, BRI, or BMI with CVD (*P* > 0.05) (Table 2).

**Table 2:** Logistic regression analysis of TyG and physical measurement indicators with incidence of CVD.

Characteristics	Crude model		Model 1		Model 2	
	OR (95% CI)	*P*-value	OR (95% CI)	*P*-value	OR (95% CI)	*P*-value
TyG index	2.113 (1.653,2.720)	<0.001	2.161 (1.603,2.940)	<0.001	2.093 (1.532,2.889)	<0.001
WtHR (per 1% increase)	1.022 (0.992,1.053)	0.152	1.009 (0.977,1.043)	0.575	1.010 (0.977,1.044)	0.570
BRI	1.233 (1.065,1.429)	0.005	1.165 (0.993,1.367)	0.061	1.167 (0.993,1.373)	0.061
ABSI (per 1% increase)	1.818 (1.473,2.256)	<0.001	1.769 (1.420,2.218)	<0.001	1.791 (1.432,2.256)	<0.001
BMI	1.044 (0.984,1.107)	0.154	1.035 (0.971,1.103)	0.294	1.028 (0.964,1.097)	0.407

OR, odds ratio; CI, confidence interval.

The crude model adjusted for none.

Model 1 adjusted for sex, age, hypertension, diabetes, family history of cardiovascular or cerebrovascular disease, SBP, and DBP.

Model 2 adjusted for Model 1 + WBC, eGFR, neutrophil, and HDL-C.

**Figure 2:**
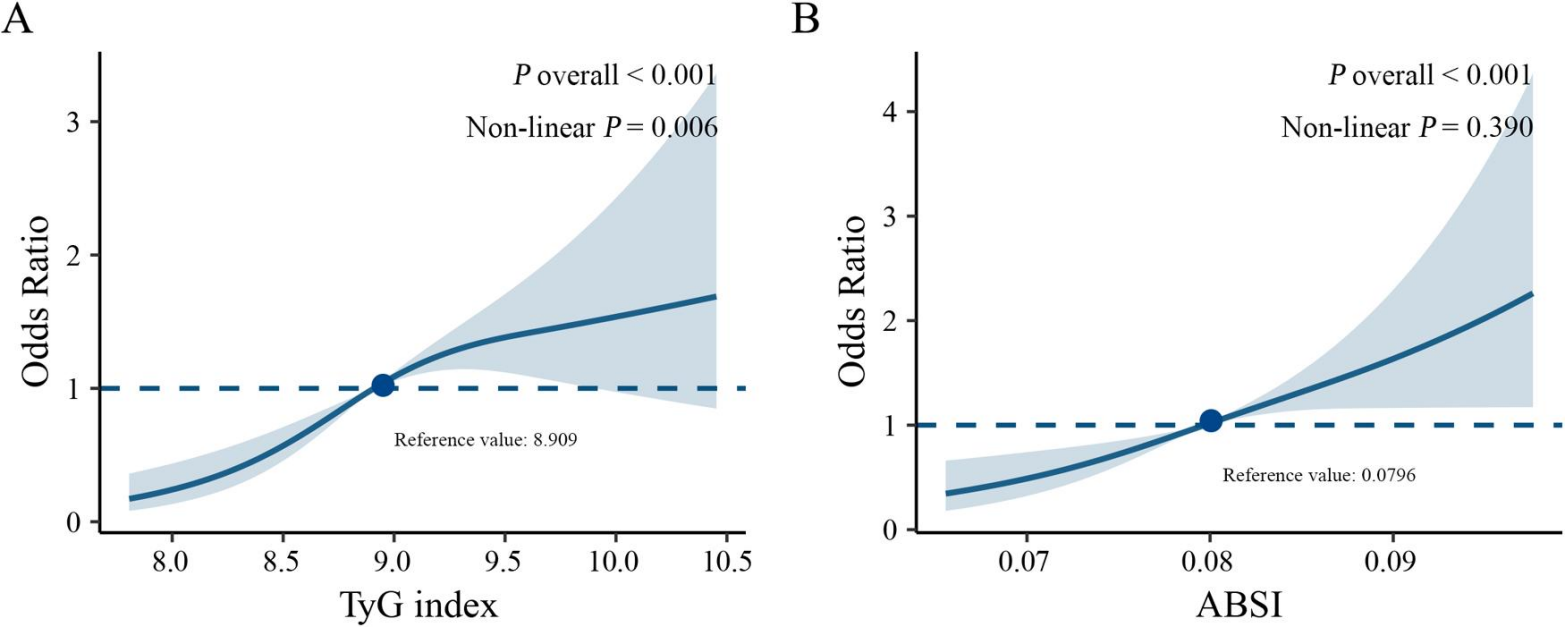
Restricted cubic spline curves showing the association between TyG index and ABSI and incident CVD. **(A)** TyG index; **(B)** ABSI. Adjusted for sex, age, hypertension, diabetes, family history of cardiovascular or cerebrovascular disease, SBP, DBP, WBC, eGFR, neutrophil, and HDL-C.

RCS analysis demonstrated an S-shaped relationship between the TyG index and incident CVD (*P* for nonlinearity = 0.006), while ABSI showed a linear association with incident CVD (*P* for nonlinearity = 0.390) (Figure 2).

**Figure 3:**
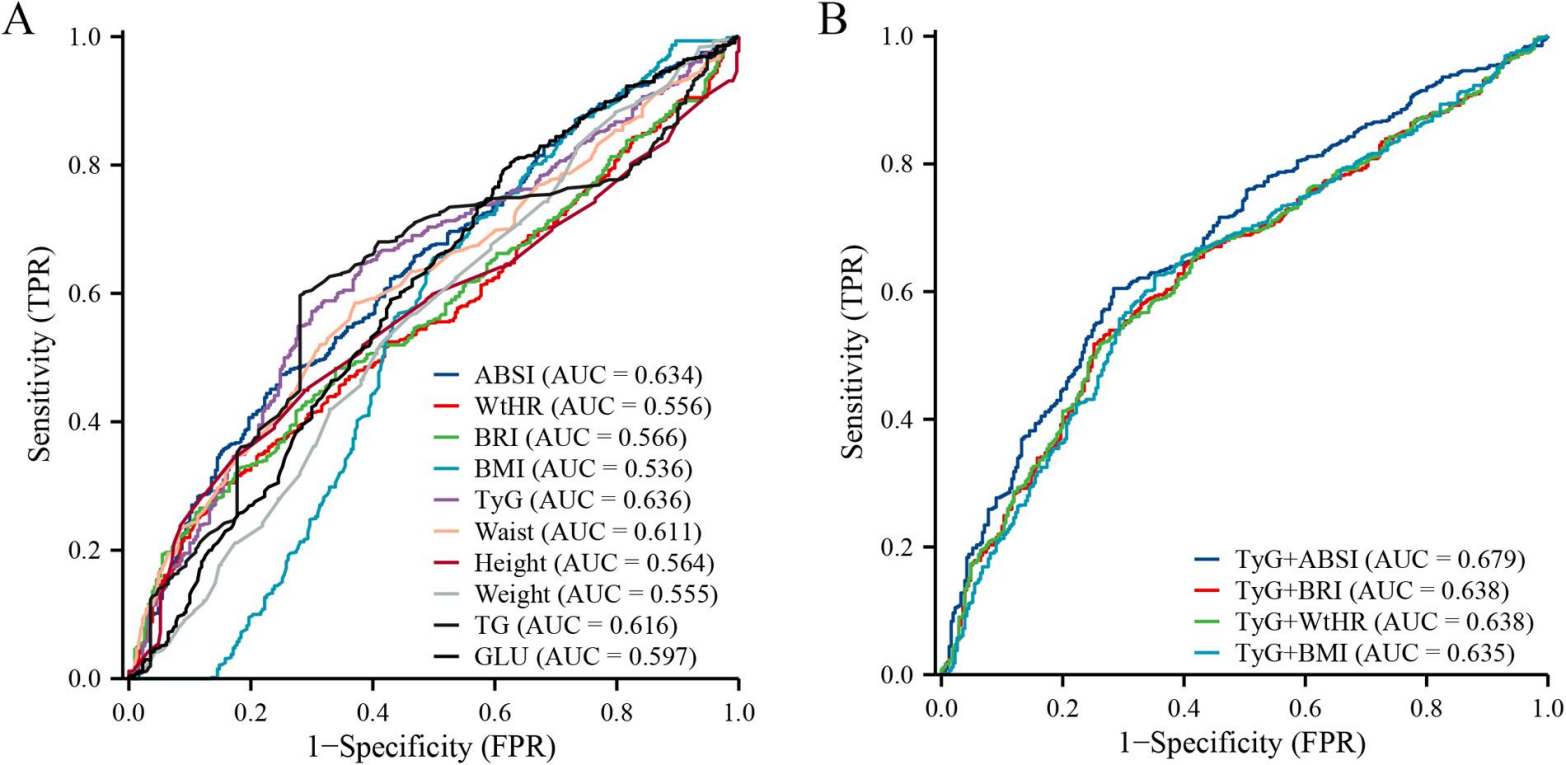
Receiver operating characteristic curve for predicting CVD in overweight and obese patients. **(A)** Individual indicators predict CVD; **(B)** TyG index combined with physical measurements predicts CVD.

ROC analysis indicated that the TyG index (AUC: 0.636, 95% CI: 0.598–0.673), ABSI (AUC: 0.634, 95% CI: 0.597–0.671), WC (AUC: 0.611, 95% CI: 0.574–0.648), and TG (AUC: 0.616, 95% CI: 0.578–0.654) exhibited fair predictive ability for CVD (Figure 3A). For TyG, the optimal cut-off value was 8.881, corresponding to a sensitivity of 0.572 and a specificity of 0.700, with a positive predictive value (PPV) of 0.790, a negative predictive value (NPV) of 0.454, and a Youden index of 0.272. For ABSI, the optimal cut-off value was 0.078, yielding a sensitivity of 0.474, specificity of 0.752, PPV of 0.790, NPV of 0.421, and a Youden index of 0.225. Further analyses of the combined models confirmed that TyG + ABSI (AUC: 0.679, 95% CI: 0.644–0.715) provided the strongest discriminative ability (Figure 3B). At the optimal probability cut-off value of 0.320, this model achieved a sensitivity of 0.605 and a specificity of 0.716, with a PPV of 0.807, an NPV of 0.479, and the highest Youden index (0.321), indicating the most favorable balance between sensitivity and specificity. In comparison, TyG + BRI, TyG + WtHR, and TyG + BMI yielded lower AUC values of 0.639, 0.638, and 0.635, respectively. These models exhibited sensitivities ranging from 0.518 to 0.625, specificities from 0.648 to 0.739, PPVs from 0.778 to 0.796, NPVs from 0.438 to 0.467, and Youden indices between 0.254 and 0.273. Overall, TyG + ABSI consistently demonstrated superior predictive performance across multiple diagnostic metrics for incident CVD (Table 3).

**Table 3:** ROC analysis of TyG and physical measurement indicators independently and in combination for predicting incidence of CVD.

Characteristic	AUC (95% CI)	Cut-off value	Sensitivity	Specificity	Positive Predictive Value	Negative Predictive Value	Yorden Index
Independent indicators							
ABSI	0.634 (0.597–0.671)	0.078	0.474	0.752	0.790	0.421	0.225
WtHR	0.556 (0.518–0.594)	0.508	0.302	0.848	0.797	0.382	0.150
BRI	0.566 (0.528–0.603)	3.519	0.266	0.884	0.818	0.380	0.149
BMI	0.536 (0.492–0.580)	26.689	0.656	0.500	0.721	0.425	0.156
TyG	0.636 (0.598–0.673)	8.881	0.572	0.700	0.790	0.454	0.272
Waist	0.611 (0.574–0.648)	92.300	0.585	0.629	0.756	0.435	0.214
Height	0.564 (0.527–0.601)	168.250	0.346	0.826	0.796	0.391	0.172
Weight	0.555 (0.515–0.595)	77.975	0.525	0.584	0.713	0.384	0.108
TG	0.616 (0.578–0.654)	1.825	0.597	0.719	0.807	0.475	0.316
GLU	0.597 (0.557–0.636)	5.845	0.803	0.374	0.716	0.492	0.177
Combined indicators							
TyG + ABSI	0.679 (0.644–0.715)	0.320	0.605	0.716	0.807	0.479	0.321
TyG + BRI	0.639 (0.603–0.676)	0.318	0.554	0.700	0.784	0.444	0.254
TyG + WtHR	0.638 (0.601–0.675)	0.310	0.518	0.739	0.796	0.438	0.257
TyG + BMI	0.635 (0.598–0.672)	0.333	0.625	0.648	0.778	0.467	0.273

For independent indicators, cut-off values represent the optimal thresholds of the corresponding variables. For combined indicators, cut-off values indicate the predicted probability thresholds (%) derived from multivariable logistic regression models, as determined by the Youden index.

**Figure 4:**
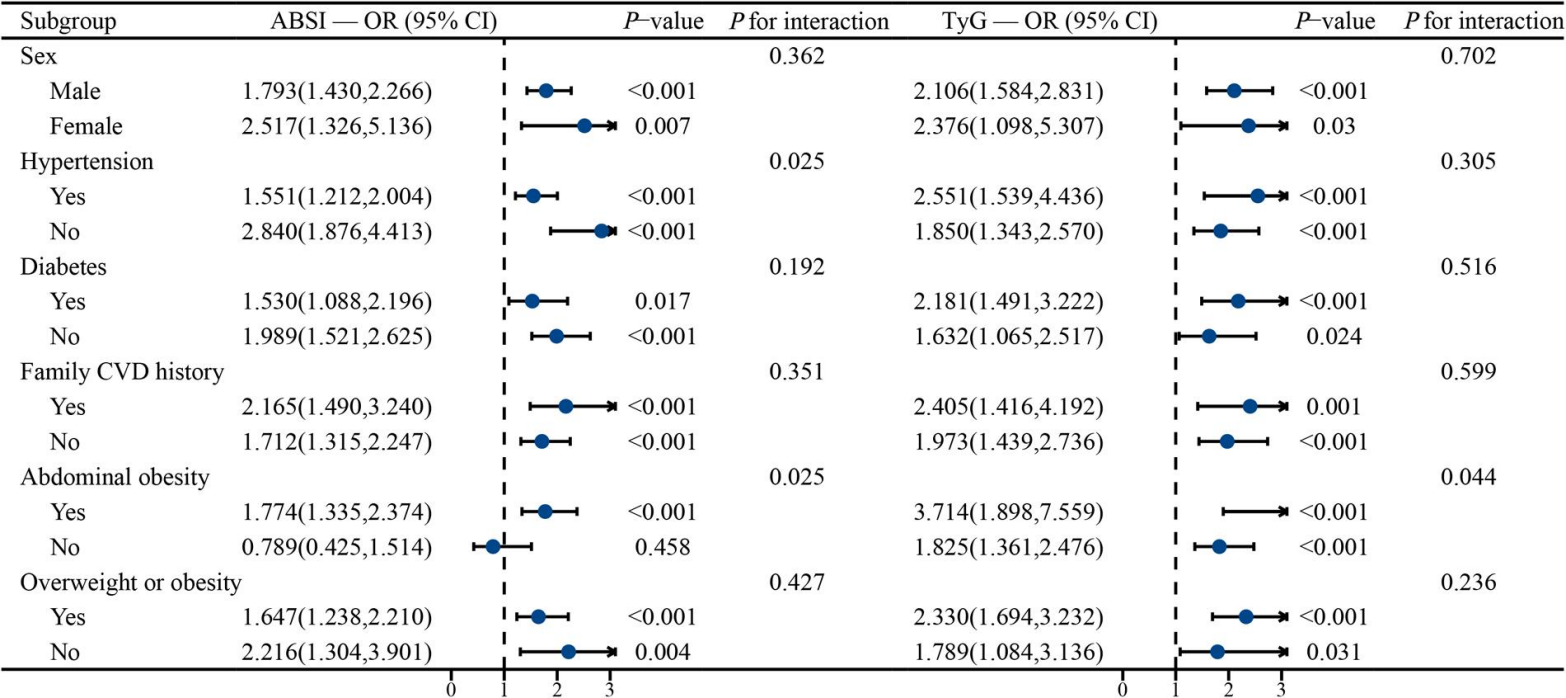
Subgroup analysis of the association between the TyG index, the ABSI, and incident cardiovascular disease (CVD). Adjusted for SBP, DBP, WBC, eGFR, neutrophil, and HDL-C.

Subgroup analyses further indicated that both the ABSI and TyG indices were associated with incident CVD across all subgroups—including by sex, diabetes, hypertension, family history of cardiovascular disease, and overweight or obesity (*P* < 0.05)—and no interactions were observed (interaction *P* > 0.05). Notably, no association between ABSI and CVD was observed in individuals without abdominal obesity (OR: 0.789; 95% CI: 0.425–1.541, *P* = 0.458) (Figure 4).

## 4 Discussion

In this study involving 920 overweight and obese Chinese adults, both the TyG index and ABSI were found to be associated with CVD. The TyG index demonstrated a nonlinear S-shaped relationship with CVD risk, whereas ABSI exhibited a linear association. Moreover, the combination of ABSI and TyG outperformed other anthropometric or laboratory indicators in predicting CVD.

Our findings are generally consistent with previous studies on the role of anthropometric indices and metabolic markers in cardiovascular risk prediction. Concerning the TyG index, Epidemiological studies across different populations have confirmed its strong associations with metabolic syndrome, type 2 diabetes, and arterial stiffness (20, 21). A large prospective cohort study in Korea reported that higher TyG index levels were significantly associated with an increased incidence of myocardial infarction and stroke, independent of traditional cardiometabolic risk factors (22). Similarly, evidence from Chinese cohort studies indicated that the TyG index could predict the prognosis of CVD outcomes such as ischemic heart disease, heart failure, and myocardial infarction (23, 24). The TyG index has been validated as a surrogate marker of IR. An elevated TyG index typically reflects greater degrees of IR (25). IR is defined as a pathological state in which peripheral tissues—including skeletal muscle, adipose tissue, and the liver—exhibit diminished biological responsiveness to normal circulating concentrations of insulin (26). Under IR, skeletal muscle fails to efficiently take up glucose, while pancreatic *β*-cells increase insulin secretion, resulting in hyperinsulinemia. In this setting, the liver continues to produce glucose despite hyperinsulinemia. Although this compensatory state can maintain euglycemia in the short term, it imposes substantial stress on *β*-cell function and initiates a cascade of metabolic disturbances (27). IR is the central pathological defect in type 2 diabetes and also exerts wide-ranging effects on cardiovascular health through mechanisms including endothelial dysfunction, dyslipidemia, hypertension, chronic inflammation, and oxidative stress (28). Mechanistically, IR reduces insulin-mediated nitric oxide (NO) synthesis and enhances vasoconstrictor activity, leading to impaired vasodilation and increased arterial stiffness. In addition, IR promotes sympathetic nervous system activation and renal sodium reabsorption, which contribute to elevated blood pressure. IR is also associated with elevated triglycerides, reduced HDL-C, and a greater proportion of small dense LDL particles, all of which accelerate the progression of atherosclerosis (29). Therefore, the strong association observed in this study between the TyG index and CVD risk is biologically plausible and consistent with the central role of IR in cardiometabolic disease.

ABSI was developed by Krakauer et al. as a mathematical model based on WC and BMI to predict mortality risk in Western populations (10). Studies have shown that ABSI is statistically independent of BMI and is not affected by the “obesity paradox,” thereby providing a more accurate reflection of abdominal obesity (30). The observed discordance between BMI-defined overweight/obesity and WC-defined abdominal obesity highlights the limitations of BMI in capturing body fat distribution. Individuals with normal BMI but excess visceral adiposity may be misclassified as metabolically low risk when BMI alone is used. The higher prevalence of abdominal obesity among participants with CVD despite a lower proportion of BMI-defined overweight/obesity supports the notion that central adiposity may confer cardiovascular risk independent of overall body mass. Abdominal obesity represents the disproportionate accumulation of fat in the abdominal region. Unlike subcutaneous adipose tissue, which is located beneath the skin, most abdominal fat consists of visceral adipose tissue (VAT), which is deposited within the abdominal cavity and surrounds internal organs such as the liver, pancreas, and intestines (31). VAT functions as an endocrine organ, secreting a variety of bioactive substances, including free fatty acids, adipokines, and pro-inflammatory cytokines (32). Previous epidemiological studies have shown that ABSI can predict diseases associated with abdominal obesity, such as colorectal cancer (33), abdominal aortic calcification (34), and type 2 diabetes (35). Further evidence has indicated that ABSI is also closely associated with the development and progression of cardiovascular disease (12). The chronic low-grade inflammatory and oxidative stress state induced by VAT contributes to endothelial dysfunction and the formation of atherosclerosis. The metabolically active nature of VAT also promotes IR and dyslipidemia, thereby increasing plaque instability and the risk of rupture. A study conducted in Chinese patients with type 2 diabetes demonstrated that ABSI was effective in predicting the progression of carotid plaques (36). Subgroup analyses in this study indicated that no significant association was observed between ABSI and CVD in individuals without abdominal obesity. As previously mentioned, ABSI is primarily used as a measure of abdominal obesity; although BMI may indicate overweight status, fat distribution is mainly concentrated in the subcutaneous fat region, and subcutaneous fat has a relatively minor impact on CVD risk. This may explain why ABSI did not show a clear association in this subgroup.

The present study showed that combining ABSI, which reflects abdominal fat accumulation and visceral adipose distribution, with the TyG index, a surrogate marker of IR, yielded higher predictive accuracy for CVD than either measure alone or in combination with other anthropometric indicators. As discussed above, ABSI reflects the anatomical burden of VAT, whereas the TyG index reflects the degree of metabolic disturbance driven by IR. Both independently increase CVD risk, but their convergence may identify individuals at particularly high risk—namely, those with excess VAT together with pronounced IR (37). Evidence from a U.S. population-based study supports our findings, showing that the combination of TyG and ABSI outperformed TyG with WtHR or BMI in predicting cardiovascular mortality (12). Furthermore, studies in Chinese populations have indicated that TyG combined with ABSI could detect stroke risk earlier than conventional anthropometric measures in the general population (17).

Notably, by quantitatively comparing the predictive performance of the TyG–ABSI combination with that of the TyG index combined with other commonly used anthropometric measures, and by focusing on incident CVD events rather than mortality outcomes, the present study provides new insights into cardiovascular risk prediction. In addition, our findings offer clinically meaningful evidence for risk stratification of incident CVD among individuals at high cardiovascular risk, namely those who are overweight or obese—a population that has often been underrepresented or insufficiently emphasized in previous studies. From a clinical perspective, the combined use of ABSI and the TyG index has practical advantages, as both indices can be readily obtained from routine anthropometric and laboratory measurements without the need for advanced imaging or costly testing. While their discriminative performance alone may not be sufficient for standalone risk prediction, these easily accessible measures may serve as useful adjuncts to existing cardiovascular risk assessment strategies, particularly in large-scale screening settings and primary care populations with overweight or obesity.

This study has several limitations. First, the participants were restricted to overweight and obese Chinese adults, which may limit the generalizability of the findings to other ethnic groups or individuals with normal weight. Second, although adjustments were made for several potential confounding factors, residual confounding due to unmeasured variables, such as dietary intake and socio-economic status, cannot be excluded. Third, because participants self-selected the timing of their health examinations during follow-up, the duration of follow-up was variable and could not be precisely determined. As a result, standard time-to-event analyses were not feasible, and logistic regression was used to model cumulative incident CVD occurrence over an unspecified follow-up window. Consequently, relative risk estimates could not be translated into absolute risks, and formal assessment of model calibration and time-dependent risk prediction was limited. Fourth, information on medication use was not available in our dataset, including baseline use or follow-up changes in lipid-lowering agents, antidiabetic medications, antiplatelet therapy, and other cardiovascular preventive treatments. In particular, the use of medications that may influence insulin resistance, glucose and triglyceride metabolism, or body composition—such as metformin, thiazolidinediones, fibrates, high-dose omega-3 fatty acids, glucagon-like peptide-1 receptor agonists, and sodium-glucose cotransporter-2 inhibitors—was neither used as an exclusion criterion nor adjusted for in the statistical models. Because these treatments may affect TyG index, anthropometric parameters, and cardiovascular risk, residual confounding related to medication exposure cannot be excluded. Finally, the study population was derived from a real-world health examination cohort, in which men were substantially overrepresented. This sex imbalance reflects the characteristics of the source population rather than the study design itself and may affect the applicability of the findings to female populations.

## 5 Conclusion

In overweight and obese Chinese adults, both ABSI and the TyG index were independently associated with the prevalence of CVD. Their combined use demonstrated moderate discriminative ability, suggesting potential value in identifying individuals with an unfavorable cardiometabolic risk profile. Given their simplicity and low cost, ABSI and the TyG index may serve as promising, easily obtainable complementary markers for cardiovascular risk assessment. However, further external validation and direct comparison with established multivariable risk scores are warranted before these indices can be considered for routine clinical application.

## Data Availability

The raw data supporting the conclusions of this article will be made available by the authors, without undue reservation.
