# Supplementary material for: A body shape index combined with the triglyceride-glucose index for cardiovascular risk prediction in overweight and obese Chinese adults
Source: Front Cardiovasc Med. 2026 Apr 29;13:1712006. doi: 10.3389/fcvm.2026.1712006 (PMC13167947; doi:10.3389/fcvm.2026.1712006)
Supplement: Supplementary file 1 [file Table1.docx]

Supplementary Table 1: Number and proportion of missing covariates

| Variables | Number of missing | Proportion |
| --- | --- | --- |
| Uric acid | 223 | 24.2% |
| eGFR | 59 | 6.41% |
| albumin | 47 | 5.11% |
| Glucose | 39 | 4.24% |
| LDL-C | 26 | 2.83% |
| ALT | 21 | 2.28% |
| AST | 21 | 2.28% |

Note: This study included a total of 920 patients, and multiple imputation methods were employed to address the aforementioned missing values. Uric acid was excluded due to a significant number of missing values.
